# Some Things for Dentists to Think about

**Published:** 1908-07-15

**Authors:** S. E. Dodson

**Affiliations:** Grand Rapids, Mich.


### SOME THINGS FOR DENTISTS TO THINK ABOUT.

BY S. E. DODSON, D.D.S., GRAND RAPIDS, MICH.

Read before the Southwestern Michigan and Fifth District Dental Societies, Grand Rapids, Mich., April 15, 1908.

"Discord is harmony misunderstood."

We know what appeals to our faculties as music. We are therefore ready to detect any jar or departure from harmony the moment it reaches our ear. We know that elementally there is nothing but sweetness and beauty. In this particular collection of sounds and with a different and proper arrangement those same sounds would produce beautiful tones, in short, "harmony."

It is very much the same, then, in orthodontia. Malocclusion is occlusion misplaced. To the orthodontist belongs the task of rearranging those elements, the teeth. I am speaking to you as dentists, from, of course, an orthodontist's standpoint.

I do not believe that a dental meeting is a fit or proper place to read papers on the treatment of the various classes of malocclusion. The dentists are not, nor do they want to be orthodontists. The up-to-date dentist sends his cases to the orthodontist, and holds him responsible for the proper treatment of the same, with no worry or bother to himself.

Dentistry is a specialty of medicine. Physicians do not attempt to cast gold inlays, removable bridge work, prophylaxis, or any of the newer operations in Dentistry. Therefore the dentist who has only the best interests of his patients at heart, keeps out of the field of orthodontia, which is a specialty of dentistry. I do not say all this because Iwant in my work is an orthodontist, your cases to do. There are other fields from which we can gather our work, and I can say for myself, with all honesty, that I do not care if

I never receive another case of orthodontia from a dentist.

With the rhinologist, the physician, the teacher in the public schools and the parents themselves all on the lookcut for the welfare for these little patients, the dentist is far from being " the whole thing." The dentist in his capacity of dental advisor to the family, should be the first to discover and warn his patients of the necessity for treatment and it is with the object of stimulating him in this direction that this is written.

He should get and read Dr. Angle's book on malocclusions of the teeth. He should learn the different classes of irregularity. He should know the difference between these classes. It would be well for him to be able to distinguish between cases wherein the maxilla was merely under-developed, as in many cases of Class 1, and an over-developed mandible, as in Class 3; the facial outline in the former being much the same as the latter.

I could go on at much length on this same topic, but do not wish to take your time. It is to be understood that this is said from the standpoint of an orthodontist. It is for the purpose of calling your attention to what appeals to me as facts, which if not known, serve to put the dentist in a very awkward position, many times, when questioned by parents as to what the treatment of the orthodontist is likely to consist of, and its probable results facially.

I would recommend to your notice an article on mouth-breathing, by Dr. Woods Hutchinson, published in the Saturday *Evening Post*, of April 4. A careful perusal of this will go further to arouse an interest in your minds, no doubt, than anything I would be capable of writing, or telling you. For the benefit of those who cannot get a copy of the Post, if they will give me their names I will be glad to mail it to him, as I have twenty or thirty copies which I bought for my patients.

This paper is necessarily brief and I think you will like it all the better for that. The discussion which I feel it should bring forth, will, I think, prove beneficial to all.

### DISCUSSION.

Dr. C. B. Blackmarr: I am very glad that Dr. Dodson brought out the fact about Dr. Wood Hutchinson's article in the Saturday *Evening Post.* I think that is my point at this meeting, to try and have a whole lot of these copies distributed and to have shown to us the responsibility for our patients' general health that we are not recognizing.

Dr. Hoff: There is another phase of the subject that I would like to speak about, but I don't want to take all the time. Dr. Dodson's paper, as I gathered from the reading of it, is a plea for specialists or specialism. Now I most cordially agree with that idea. I believe that the time has come when we must specialize, whether we want to or not. Technically, perhaps, some of you who do not keep up to date on the advances that are being made, do not see the burden that is being put on the average dentist of to-day the way I see it. I have been one of those people who have always prided myself on trying to keep up with everything that is being done. Everything that comes up that is new I try to learn all about it that I can. I find that I cannot do it, however. It is practically impossible for any dentist to-day, and especially a man who is in full practice, as you all are, to keep up with all the advances that are being made in dentistry. No man can do it and do it as it ought to be done, so I would most cordially commend the man who has the courage to specialize and tries to do his work worthily, I don't care whether it is orthodontia, or what it is, such a man ought to be encouraged. We ought to help him, in fact, and we ought to feel it our duty to refer patients, that we cannot properly handle, to men who are willing to withdraw themselves from general practice and take up a special line of work. The results of which, so far as money is concerned, are often problematical. I feel as though the time is coming when more of this specializing in different branches of the work has got to come, and when men do undertake to specialize in anything in our profession, it ought to meet with our approval and our encouragement.

Dr. Runyan: The relation that dentists should hold as between the parents and children and our own specialist and the family physician is a growing problem of great interest and importance. Now we cannot all specialize on all these subjects, but we should be intelligent so that when we can see a case requiring special treatment and know where to place it. The child's teeth should be taken care of from the time they begin to erupt, and the dentist is the only one that is prepared to recognize true conditions. The dentist, if he is as intelligent as he should be, will recognize as soon as he looks into a child's mouth, whether there are adenoids, or whether enlarged tonsils, and he should be in such relation with parents that they have confidence enough in him to rely on his judgment and also to rely on his recommendation. When the tonsils are enlarged or when there are adenoids, it is our duty to recommend to the parents of our child patients that they be taken to the rhinologist, if there are adenoids, or to a throat specialist to have the tonsils removed, and explain thoroughly to the parents what the results will be if these conditions are not taken care of. All this brings us into proper relation with the physician, the throat and lung specialist, as well as our own specialists. Each one's responsibility is not entirely removed by just doing the work that just comes to his hand, but we should all work in harmony so that we can all be working together, that the patient may receive the best results from working together.

Dr. F. E. Williams: I agree upon the points mentioned, and I hope your attention may be drawn more closely to observing occlusion of the teeth. This matter of occlusion should bind us together as a whole, the specialist and the general practitioner. And we must all be prepared to give light to any patient. As some one brought out yesterday, our patients are our kindest teachers; they want to know the conditions, and the reason why, and it devolves upon us to be able to tell them. And as the doctor who has just spoken says, our relations between ourselves and different specialists in medicine, we should be able to recognize the conditions that would naturally come under their treatment, and be somewhat cognizant of the different treatments involved, to be able to intelligently direct our patients in order that they may receive the benefits that they should have, and at the right time, at the beginning stage. We orthodontists must get at our patients sooner than we do, as then the trouble or difficulty is in its inception and they can be most readily corrected.

DR. DODSON: There is only one point that was brought up in the discussion that I care to speak of particularly and that is one of Dr. Hoff's remarks. I cannot let him get away with the idea, or have you folks get away with the idea, that this little article was written with a view to benefit specialists, that is to benefit orthodontists. I am perfectly sincere in saying that it is for the benefit of the dentists themselves. You all know, the men who have given the subject any attention at all, how many of these cases there are that go through your hands continually, and those of you who don't know had better find out, because they are going through your hands every day, and all of you have a number of these patients now. This paper was written simply with a view of awakening your interest on that subject. The matter as to whether or not dentists send me, personally, a case of orthodontia, I meant just what I said, doesn't make a particle of difference. I speak impersonally for the good of the cause as I see it. Orthodontia is launched and it is going to stay launched. As to Dr. Hoff's further remarks about a man trying to cover the entire field, I agree with him. We are in the position of the man who had a horse which was a "wind sucker." He advised his man to keep the manger full of hay, and when he inquired next day how he was coming on, the stableman replied, "God, no man can do it." Dr. Runyan has given my ideas perfectly, as to public work. Having had the pleasure of seeing a few of his slides, I think he is engaged in about the noblest work of any of us. I only wish I had the courage to undertake the work myself.

